# Cancer Marker Immunosensing through Surface-Enhanced Photoluminescence on Nanostructured Silver Substrates

**DOI:** 10.3390/nano13243099

**Published:** 2023-12-07

**Authors:** Georgia Geka, Anastasia Kanioura, Ioannis Kochylas, Vlassis Likodimos, Spiros Gardelis, Anastasios Dimitriou, Nikolaos Papanikolaou, Kalliopi Chatzantonaki, Ekaterina Charvalos, Anastasios Economou, Sotirios Kakabakos, Panagiota Petrou

**Affiliations:** 1 Immunoassays/Immunosensors Lab, Institute of Nuclear & Radiological Sciences & Technology, Energy & Safety, NCSR “Demokritos”, 15341 Aghia Paraskevi, Greece; g.gkeka@rrp.demokritos.gr (G.G.); nkanioura@ipta.demokritos.gr (A.K.); skakab@rrp.demokritos.gr (S.K.); 2 Department of Chemistry, National and Kapodistrian, University of Athens, University Campus, 15771 Athens, Greece; aeconomo@chem.uoa.gr; 3 Section of Condensed Matter Physics, Department of Physics, National and Kapodistrian University of Athens, University Campus, 15784 Athens, Greece; ikochyla@phys.uoa.gr (I.K.); vlikodimos@phys.uoa.gr (V.L.); sgardelis@phys.uoa.gr (S.G.); 4 Institute of Nanoscience & Nanotechnology, NCSR “Demokritos”, 15341 Aghia Paraskevi, Greece; a.dimitriou@inn.demokritos.gr (A.D.); n.papanikolaou@inn.demokritos.gr (N.P.); 5 Molecular Diagnosis Department, INVITROLABS S.A., 12251 Peristeri, Greece; p.chatzantonaki@invitrolabs.gr (K.C.); directorcentralabs@iaso.gr (E.C.)

**Keywords:** optical biosensor, ovarian cancer, CA125, HE4, immunochemical detection, photoluminescence

## Abstract

Nanostructured noble metal surfaces enhance the photoluminescence emitted by fluorescent molecules, permitting the development of highly sensitive fluorescence immunoassays. To this end, surfaces with silicon nanowires decorated with silver nanoparticles in the form of dendrites or aggregates were evaluated as substrates for the immunochemical detection of two ovarian cancer indicators, carbohydrate antigen 125 (CA125) and human epididymis protein 4 (HE4). The substrates were prepared by metal-enhanced chemical etching of silicon wafers to create, in one step, silicon nanowires and silver nanoparticles on top of them. For both analytes, non-competitive immunoassays were developed using pairs of highly specific monoclonal antibodies, one for analyte capture on the substrate and the other for detection. In order to facilitate the identification of the immunocomplexes through a reaction with streptavidin labeled with Rhodamine Red-X, the detection antibodies were biotinylated. An in-house-developed optical set-up was used for photoluminescence signal measurements after assay completion. The detection limits achieved were 2.5 U/mL and 3.12 pM for CA125 and HE4, respectively, with linear dynamic ranges extending up to 500 U/mL for CA125 and up to 500 pM for HE4, covering the concentration ranges of both healthy and ovarian cancer patients. Thus, the proposed method could be implemented for the early diagnosis and/or prognosis and monitoring of ovarian cancer.

## 1. Introduction

Nanomaterials have found applications in the biosensor field both as solid supports that provide a high surface-to-volume ratio and mechanical strength, and labels that lead to signal amplification, thus improving the analytical performance of biosensors [1,2]. In particular, regarding optical biosensors, the implementation of plasmonic nanostructured materials as substrates has continuously gained ground over the last few decades [3,4,5,6]. Nanostructured noble metals such as gold (Au) and silver (Ag) are characterized by enhanced light absorption and/or scattering, which can be controlled by altering their structure geometry and size, causing a dramatic increase in the optical signal compared to non-plasmonic materials [7,8]. For the fabrication of these structures, several methods have been employed depending on their composition and the desired micro- and nano-structuring. These methods include, for example, lithographic techniques, chemical vapor deposition, and metal-assisted chemical etching [9,10,11,12].

The optical biosensors that have been developed based on such structures employ different transduction principles, such as photoluminescence (PL), interferometry, surface-enhanced Raman scattering (SERS), and surface plasmon resonance (SPR) [13,14]. Photoluminescence is a form of light emission spectroscopy which follows the absorption of light by a material that excites electrons to higher energy states (excitation). When the electrons return to their ground state (relaxation), the energy can be released in the form of emitted light as fluorescence or phosphorescence, depending on the time interval between excitation and emission. In the case the excitation–emission process takes place on a nanostructured noble metal substrate, the enhanced electromagnetic field near the metallic nanostructure regulates the electron transition properties of luminescent molecules affecting the luminescent emission spectrum intensity [15,16]. The outcome is the enhancement of the emitted photoluminescence signal and therefore the increased detection sensitivity of luminescent molecules [17,18].

**Figure 1:**
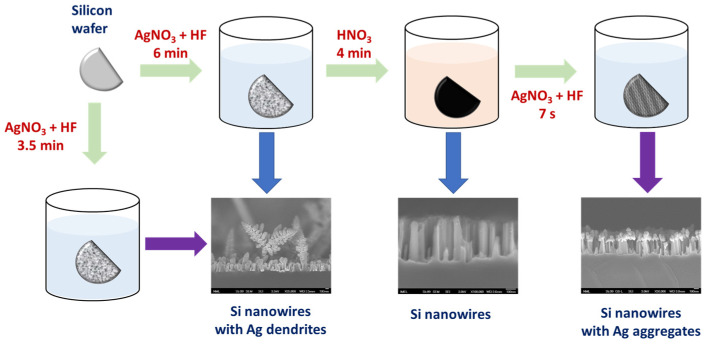
Schematic of the MACE procedure steps and representative SEM images of surfaces obtained after each step.

To this end, the aim of this work was to take advantage of nanostructured silicon surfaces decorated with silver nanoparticles as substrates for immunochemical detection through the photoluminescence of two cancer markers, namely carbohydrate antigen 125 (CA125) and human epididymis protein 4 (HE4). The substrates were fabricated by employing metal-assisted chemical etching (MACE), which is a simple procedure utilized to create, in a single step, nanowire structures on a silicon wafer with Ag dendrite structures on top of them [19,20,21] (Figure 1). The substrates can be used *as prepared* or treated in an acidic solution to remove the Ag dendrites and create aggregates of Ag nanoparticles by post-immersion in the etching/deposition solution (Figure 1). Both types of substrates have been exploited for the detection of biomolecules through surface-enhanced Raman spectroscopy [19,20], and their potential for enhancement of photoluminescence has been demonstrated using directly adsorbed fluorescent labels [21]. In this work, the two types of nanostructured surfaces were evaluated with respect to the immunochemical detection of CA125 and HE4, two ovarian cancer markers, aiming at the development of a system that could determine both analytes simultaneously in human serum samples.

Ovarian cancer is one of the most prevalent malignancies in women, with a poor prognosis and a fatality rate of 1.9% regarding the sum of cancer-related deaths worldwide [22]. For this reason, the detection of related biomarkers is crucial for early detection, differential diagnosis, treatment selection, and monitoring of treatment response. Integrating biomarkers into clinical practice enhances the precision and personalized management of ovarian cancer, ultimately improving patient outcome and survival rate [22,23]. During the last few decades, a large number of biomarkers have been evaluated for their ability to detect epithelial ovarian cancer at an early stage [23]. Cancer or carbohydrate antigen 125 (CA125) is one of the most commonly used biomarkers, being recognized as the “gold standard” for diagnosis and treatment response monitoring [24]. The level of CA125 in the blood serum of healthy individuals is below 35 U/mL, and it is overexpressed in patients diagnosed with ovarian cancer [25]. However, CA125 is not a specific biomarker for ovarian cancer detection, as its levels may increase in healthy individuals depending on the phase of the menstrual cycle or pregnancy and several pathological conditions, i.e., ovarian cysts, endometriosis, and hepatitis [26]. For this reason, novel biomarkers such as human epididymis protein 4 (HE4) have been investigated as potential diagnostic tools for ovarian cancer. HE4 is a low-molecular-weight glycoprotein originally found in the epithelial cells of the human epididymis [27]. HE4 is considered a more specific clinical marker, compared to CA125, for the diagnosis of ovarian cancer at an early stage and screening of the response to chemotherapy after surgery. Concentrations of HE4 in blood serum higher than 70 pmol/L (1.75 ng/mL) in pre-menopausal and 140 pmol/L (3.5 ng/mL) in post-menopausal women, respectively, indicate a high possibility of an ovarian malignancy [28]. Thus, the combination of CA125 and HE4 measurements could be used for a more efficient prediction of ovarian cancer as well as for monitoring the therapy efficiency in ovarian cancer patients [23,29]. For the immunochemical determination of these two markers, non-competitive immunoassays were developed using pairs of highly specific mouse monoclonal antibodies, one of which was used as a capture antibody and the other for detection after biotinylation. Streptavidin tagged with the fluorescent label Rhodamine Red-X was used to detect the immunocomplexes formed on the nanostructured substrates. At first, the substrates with the two different Ag nanostructures were compared, followed by an investigation of the most appropriate capture antibody immobilization method. Then, all immunoassay conditions were optimized, and the repeatability and accuracy of the assays were estimated using control samples from commercially available kits for the determination of the two analytes.

## 2. Materials and Methods

### 2.1. Materials

Hydrofluoric acid (HF; 50% in water) was obtained from Technic Inc. (Saint-Denis, France). Silver nitrate (AgNO_3_; ≥99.0%), 3-mercaptopropyl-trimethoxysilane (3-MPTMS; 95%), and N-(3-Dimethylaminopropyl)-N′-ethylcarbodiimide hydrochloride (EDC) were from Sigma-Aldrich (Darmstadt, Germany). Human cancer antigen 125 isolated from human adenocarcinoma (250 kIU) was obtained from HyTest (Turku, Finland). Recombinant human epididymis protein 4 antigen (1.94 mg/mL; ≥90%) was from EastCoast Bio (Maryland Heights, MO, USA). Mouse monoclonal antibodies against CA125 (clones 4601 and 4602 as capture and detection antibodies, respectively) and HE4 (clones 4501 and 4505 as capture and detection antibodies, respectively) were purchased from Medix Biochemica (Espoo, Finland). Bovine serum albumin (BSA) was from Acros Organics (Geel, Belgium). Streptavidin conjugated with Rhodamine Red-X or AlexaFluor 546, EZ-Link™ Sulfo-NHS-LC-Biotin (sulfo-NHS-biotin), and N-hydroxysulfosuccinimide (Sulfo-NHS) were from ThermoFischer Scientific (Waltham, MA, USA). Streptavidin conjugated with CF555 was from Biotium Inc. (Fremont, CA, USA). Lipid-stripped human serum was from Scantibodies Laboratory, Inc. (Santee, CA, USA). Twenty-four-well culture plates were from Orange Scientifique NV (Braine-l’Alleud, Belgium). All other chemicals were of analytical grade and from Merck (Darmstadt, Germany). The water used throughout the study was distilled.

### 2.2. Preparation of PL Substrates

The PL substrates were fabricated by metal-assisted chemical etching (MACE) as described in previous works [19,20] using p-type (100)-oriented monocrystalline Si wafers. Specifically, we investigated the performance of two types of substrates: one with Si nanowires decorated with Ag dendrites and the other with Si nanowires decorated with Ag aggregates. In the MACE process, the Ag dendrites are formed during the etching process of Si along with the formation of the Si nanowires, whereas the aggregates are formed by first removing the Ag dendrites, leaving the Si nanowires bare by immersion in HNO_3_ and consequently by re-immersion of the substrate with the bare Si nanowires in the initial MACE solution for a few seconds. Thus, to obtain Si nanowires decorated with Ag dendrites, the wafers were immersed into an aqueous solution containing 0.02 M AgNO_3_ and 4.8 M HF for 3.5 min. On the other hand, for Si nanowires decorated with Ag aggregates, the wafers were first immersed into the AgNO_3_/HF solution for 7 min, and then they were immersed in 50% *v*/*v* HNO_3_ for 4 min to dissolve the Ag nanostructures (dendrites) formed during the first step and leave the Si nanowires bare. Finally, they were dipped for 7 s into the aqueous AgNO_3_/HF solution to create Ag aggregates on the top of the Si nanowires. The substrates prepared following this process were characterized after each processing step by scanning electron microscopy (SEM) using a JSM-7401F SEM instrument (JEOL Europe bv; Zaventem, Belgium) operating at 30 kV. A schematic depiction of the procedure followed as well as characteristic SEM images of the Si surfaces after each processing step are provided in Figure 1. The full-size SEM images of Si nanowires decorated with Ag dendrites, bare Si nanowires after the removal of Ag dendrites, and Si nanowires decorated with Ag aggregates are provided in Figure S1. As shown, in the substrates of Ag-dendrite-decorated SiNWs, the SiNWs had lengths around 250 nm, and the Ag dendrite height exceeded 1 μm, whereas in the case of Ag-aggregate-decorated SiNWs, the SiNWs had lengths in the range of 440–480 nm (due to a longer etching time in the first step), and the silver aggregates’ size varied between 120 and 150 nm.

### 2.3. Modification of PL Substrates with 3-Mercaptopropyl-Trimethoxysilane (3-MPTMS)

The modification of PL substrates with 3-MPTMS was performed by immersion of the surfaces on a 4% (*v*/*v*) 3-MPTMS in absolute ethanol for 2 h under ambient conditions in a hood. Then, the substrates were washed with ethanol, dried under a nitrogen stream, and used immediately for antibody immobilization.

### 2.4. Biotinylation of Anti-CA125 and Anti-HE4 Antibodies

The mouse monoclonal antibodies against CA125 clone 4602 and HE4 clone 4505 were biotinylated to be used as detection antibodies in the respective non-competitive immunoassays. For this purpose, the antibody solutions were first dialyzed against a 0.25 M carbonate buffer, pH 9.2, containing 9 g/L NaCl, and then mixed with an appropriate volume of a 100 μg/mL sulfo-NHS-biotin solution in dimethyl sulfoxide so that the weight ratio of sulfo-NHS-LC-biotin to antibody in the reaction mixture would be 1:1. After incubation for 2 h at room temperature, the reaction mixture was dialyzed against 0.1 M NaHCO_3_ solution, pH 8.5, 9 g/L NaCl, 0.5 g/L NaN_3_. The biotinylated mouse monoclonal antibody solutions were kept at 4 °C.

### 2.5. Immunoassay on PL Substrates

**Figure 2:**
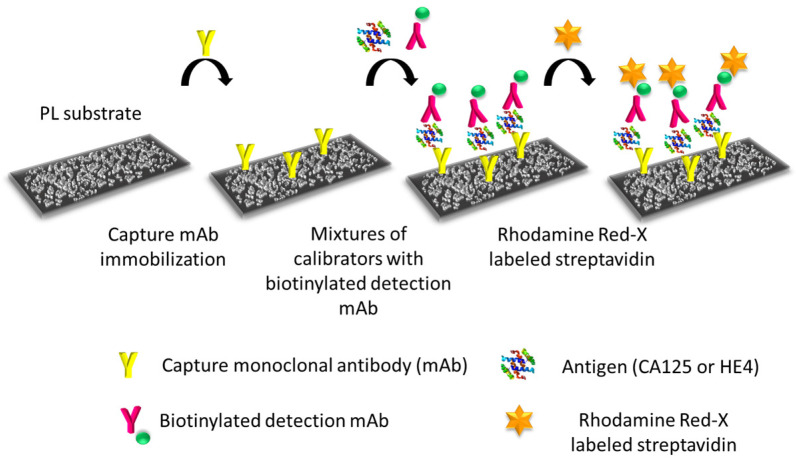
General scheme of the non-competitive immunoassay for detection of markers CA125 and HE4 by photoluminescence on the nanostructured Ag substrates.

Immunoassays were performed on substrates with dimensions of 0.5 × 0.5 cm^2^ placed onto 24-well polystyrene plates following the non-competitive immunoassay format depicted in Figure 2.

#### 2.5.1. CA125 Immunoassay

The substrates were incubated with 10 μL of a 200 μg/mL anti-CA125 mAb (clone 4601) solution in 10 mM Tris-HCl, pH 8.25, 0.1 M NaCl, overnight at 4 °C. After washing twice with 300 μL of a 10 mM Tris-HCl buffer, pH 8.25, 9 g/L NaCl (washing solution), the free binding sites of the surfaces were blocked through incubation in a 10 mg/mL BSA solution in 0.1 M NaHCO_3_, pH 8.5, for 2 h at room temperature. After washing as previously described, 200 μL of 1:1 volume mixtures of CA125 calibrators in 50 mM Tris-HCl, pH 7.8, 10 g/L BSA, 9 g/L NaCl, 0.5 g/L NaN_3_, with a 2.5 μg/mL solution of biotinylated anti-CA125 mAb (clone 4602) in the same buffer, was added per well, and the substrates were incubated for 1 h under shaking at room temperature. The substrates were washed with buffer containing 0.5 mL/L Tween 20 (3 × 300 μL), and then 200 μL of a 5 μg/mL streptavidin–Rhodamine Red-X solution in 50 mM PBS, pH 6.5, 10 g/L BSA, was added to each well and incubated for 30 under shaking at room temperature. Finally, the substrates were washed with washing solution (3 × 300 μL) and distilled water and dried prior to measurement of PL signal intensity.

#### 2.5.2. HE4 Immunoassay

The substrates were incubated with 10 μL of a 200 μg/mL anti-HE4 mAb (clones 4501) solution in 50 mM carbonate buffer, pH 9.2, overnight at 4 °C. After washing twice with 300 μL of a 10 mM phosphate-buffered saline (PBS), pH 7.4, (washing solution), the free binding sites of the surfaces were blocked through incubation in a 10 g/L BSA solution in 0.1 M NaHCO_3_, pH 8.5, for 2 h at room temperature. After washing as previously described, 200 μL of 1:1 volume mixtures of HE4 calibrators in 50 mM PBS, pH 7.4, 10 g/L, BSA, 0.5 g/L NaN_3_, with a 5.0 μg/mL solution of biotinylated anti-HE4 mAb (clone 4505) in the same buffer was added per well, and the substrates were incubated for 1 h under shaking at room temperature. The substrates were washed with washing solution containing 0.5 mL/L Tween 20 (3 × 300 μL), and 200 μL of a 5 μg/mL streptavidin–Rhodamine Red-X solution in 50 mM PBS, pH 6.5, 10 g/L BSA, was added to each well and incubated for 30 under shaking at room temperature. Finally, the substrates were washed with washing solution (3 × 300 μL) and distilled water and dried prior to measurement of PL signal intensity.

### 2.6. PL Measurements

The PL signal intensity measurements were carried out using an in-house-developed set-up that included a green diode laser (532 nm wavelength) for sample illumination at a 45-degree angle through a focusing lens (Figure S2). The diameter of the illuminated area was approximately 2 mm, and the laser power on the sample was approximately 2 mW. Under these conditions, there was no indication of heating or quenching of the photoluminescence signal intensity for illumination times up to 15 s. The emitted light was collected by an optical fiber through a long-pass filter to minimize interference from the excitation laser and directed towards a spectrophotometer (Ocean Insight; Duiven, The Netherlands). From each sample, 5 spectra were collected, and three sample replicates were used in all cases, unless otherwise indicated. For the preparation of the calibration curves, the net PL signals corresponding to each calibrator were used. Net PL signals were obtained by subtracting from the mean maximum signal of the PL spectra of each calibrator (PL value at the peak of the spectrum) the corresponding value of the zero calibrator, i.e., the signal received in the absence of the analyte.

## 3. Results

### 3.1. Selection of Substrates and Fluorescent Dyes

**Figure 3:**
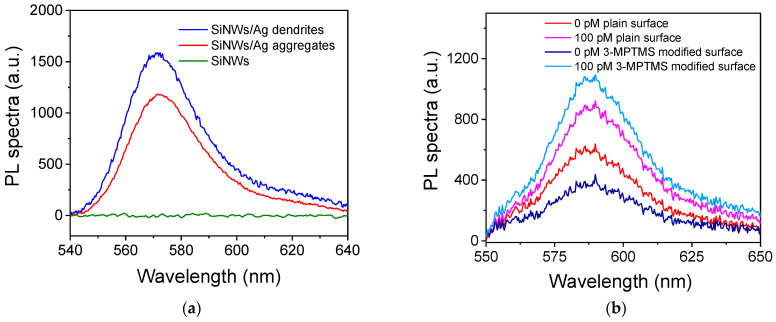
(**a**) PL spectra received from surfaces with bare SiNWs (black line) or SiNWs decorated either with Ag dendrites (red line) or Ag aggregates (blue line) for a CA125 calibrator with a concentration of 200 U/mL. (**b**) PL spectra received for the zero HE4 calibrator (red and dark-blue lines) and a calibrator containing 100 pM HE4 (magenta and light-blue lines) from plain surfaces of SiNW–Ag dendrites (red and magenta lines) or surfaces modified with 3-MPTMS (dark-blue and light-blue lines).

The substrates used in this study were prepared in silicon wafers following the MACE procedure, which results in silicon nanowires (SiNWs) decorated with either Ag dendrites or aggregates [19,20,21]. Both types of substrates were evaluated for detection through PL by performing a non-competitive immunoassay for CA125 using a biotinylated detection antibody in combination with streptavidin labeled with a rhodamine derivative (Rhodamine Red-X). For comparison reasons, a substrate with bare SiNWs was also tested. The PL spectra obtained from the three surfaces for a CA125 calibrator with a concentration of 200 U/mL are presented in Figure 3a. As shown, the surfaces with the bare SiNWs provided a negligible PL signal compared to those with SiNWs decorated with Ag nanostructures. On the other hand, comparison of SiNWs decorated with Ag dendrites and aggregates demonstrated that the former ones provided approximately 40% higher peak fluorescence signal values compared to those obtained from the Ag aggregate–SiNW substrates. Therefore, they were selected for further experimentation. It should be noted that the Ag dendrite–SiNW substrates were used up to 2 weeks after their preparation while being kept under ambient conditions without any noticeable effect on their performance.

Prior to further assay optimization, the effect of different surface treatments on the PL signal intensity was investigated. Therefore, the capture anti-HE4 antibody was immobilized onto the selected substrates by physical absorption without any treatment or after modification with a thiol-terminated silane (3-MPTMS). As shown in Figure 3b, the use of 3-MPTMS-modified surfaces resulted in an increase in the specific signal by approximately 19% with a simultaneous decrease in the non-specific signal by approximately 59%. Thus, the net signal for the 100 pM HE4 calibrator obtained from the substrates treated with 3-MPTMS increased by a total of 140% compared to the net signal obtained for the same calibrator from a substrate that had not been treated with 3-MPTMS. Thus, substrate modification with 3-MPTMS prior to the immunoassay was adopted in the final protocol.

**Figure 4:**
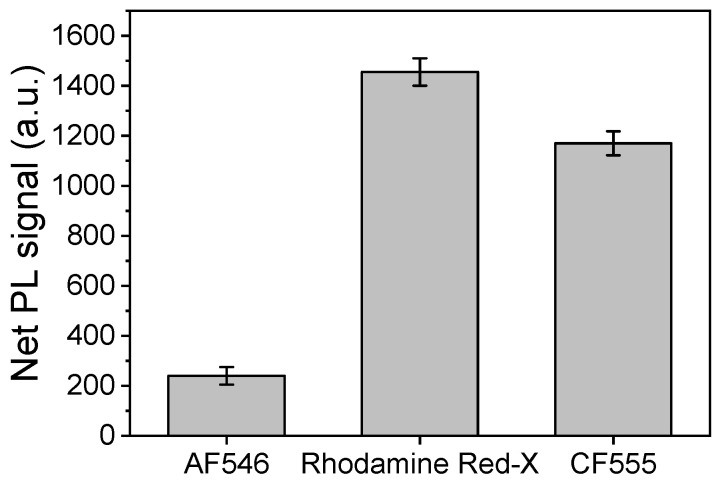
Net PL intensity values received for a 200 U/mL CA125 calibrator using streptavidin labeled with three different fluorescent dyes at a concentration of 10 μg/mL. The concentration of the capture antibody was 200 μg/mL, and that of the biotinylated detection antibody was 5.0 μg/mL. Each point is the mean value of 5 measurements from 3 replicate samples ± SD.

After the selection of the best-performing substrate, the fluorescent dye to be used was selected between three commercially available streptavidin conjugates with fluorescent labels whose excitation maximum matched the excitation laser wavelength. More specifically, the three fluorescent labels tested were Rhodamine Red-X, AlexaFluor 546 (AF546), and CF555. Amongst these three dyes, Rhodamine Red-X provided the highest signal, as shown in Figure 4 for the CA125 assay. This result can be ascribed to the higher quantum yield of this particular dye in combination with a more reactive streptavidin. Similar results were obtained for the HE4 assay.

### 3.2. Imunoassay Optimization

The immunoassays for CA125 and HE4 were separately optimized using SiNW substrates decorated with Ag dendrites and modified with MPTMS. Amongst the assay parameters optimized were the capture antibody concentration used for coating, the biotinylated detection antibody concentration, the immunoreaction duration, and the incubation time with the Rhodamine-labeled streptavidin, as well as the composition of all the solutions used, including the coating, the blocking, the washing, and the assay buffer.

**Figure 5:**
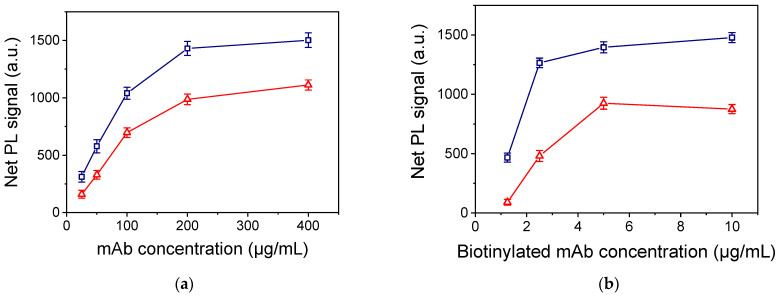
(**a**) Net PL intensity values obtained for a 200 U/mL CA125 (blue squares) and a 100 pM HE4 calibrator (red triangles) versus the concentration of respective capture antibodies in the coating solution. The biotinylated detection antibody was used at a concentration of 5.0 μg/mL for both analytes. Each point is the mean value of 5 measurements from 3 replicate samples ± SD. (**b**) Net PL intensity values obtained for a 200 U/mL CA125 (blue squares) and a 2.5 ng/mL HE4 calibrator (red triangles) versus the concentration of respective biotinylated detection antibodies. The capture antibody concentration was 200 μg/mL for both analytes. Each point is the mean value of 5 measurements from 3 replicate samples ± SD.

Regarding the coating of the substrates with the capture antibody specific for each analyte, solutions with concentration ranging from 25 to 400 μg/mL were tested. As shown in Figure 5a, the net PL signal value increased as the capture antibody in the coating solution increased and reached maximum plateau values for concentrations equal to or higher than 200 μg/mL for CA125 and HE4. Thus, this concentration was selected for both assay protocols. 

The coating solution was also selected amongst three different buffers: 50 mM phosphate buffer, pH 7.4; 10 mM Tris-HCl, pH 8.25, 0.1 M NaCl; and 50 mM carbonate buffer, pH 9.2. It was found that for the CA125 assay, the highest signals were received using 10 mM Tris-HCl, pH 8.25, and 0.1 M NaCl, whereas for the HE4 assay, the 50 mM carbonate buffer, pH 9.2, provided the highest net signals. Next, the concentration of biotinylated detection antibodies was optimized. As shown in Figure 5b, maximum plateau signal values were obtained for CA125 using the biotinylated detection antibody at a concentration of 2.5 μg/mL, while HE4 plateau values were received for a biotinylated antibody concentration of 5.0 μg/mL. The optimum composition of the assay buffer, i.e., the buffer in which the calibrators and the biotinylated antibodies solutions were prepared, was also determined. For CA125, the optimum assay buffer was 50 mM Tris-HCl, pH 7.8, 10 g/L BSA, 9 g/L NaCl, and 0.5 g/L NaN_3_, whereas for HE4, it was 50 mM PBS, pH 7.4, 10 g/L, BSA, and 0.5 g/L NaN_3_. 

**Figure 6:**
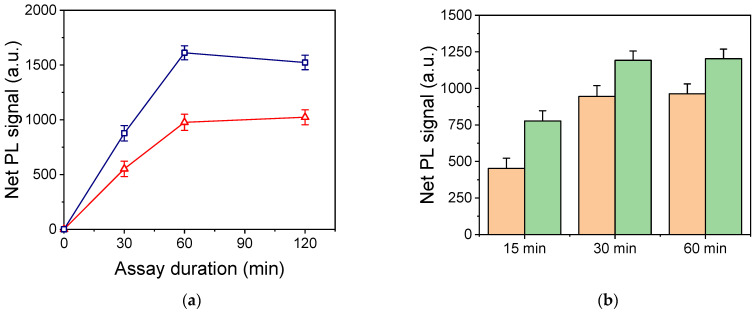
(**a**) Net PL intensity values obtained for a 200 U/mL CA125 calibrator (blue squares) and a 100 pM HE4 calibrator (red triangles) versus the duration of the immunoassay step. Each point is the mean value of 5 measurements from 3 replicate samples ± SD. (**b**) Net PL intensity values received for a 100 pM HE4 calibrator versus the reaction time with Rhodamine-labeled streptavidin solutions with a concentration of 5 (orange column) or 10 μg/mL (green columns). Each column is the mean value of 5 measurements from 3 replicate samples ± SD.

The duration of the immunoreaction step, as well as the incubation with the Rhodamine-labeled streptavidin, were also optimized. Regarding the immunoreaction duration, it was found that from 30 min to 1 h, the signal significantly increased for both analytes without a statistically significant increase in the zero calibrator signal (non-specific binding). However, as shown in Figure 6a, a further increase in the immunoreaction duration to 2 h resulted in a slight decrease (not statistically significant) in the signal for the CA125 assay, while for the HE4 assay, plateau signal values were obtained for an immunoreaction duration equal to 1 h. Therefore, 1 h was selected as the duration of the immunoreaction for both CA125 and HE4. The concentration of Rhodamine-labeled streptavidin and the reaction time with the biotinylated antibodies immobilized onto the substrates were also optimized. As shown in Figure 6b, maximum plateau signal values were obtained for both streptavidin–Rhodamine Red-Χ concentrations after 30 min of reaction. In addition, although the higher streptavidin concentration (10 μg/mL) provided approximately 25% higher signals, the concentration of 5 μg/mL was selected for the final protocol to reduce the non-specific signal as much as possible.

### 3.3. Analytical Characteristics of CA125 and HE4 PL Assays

**Figure 7:**
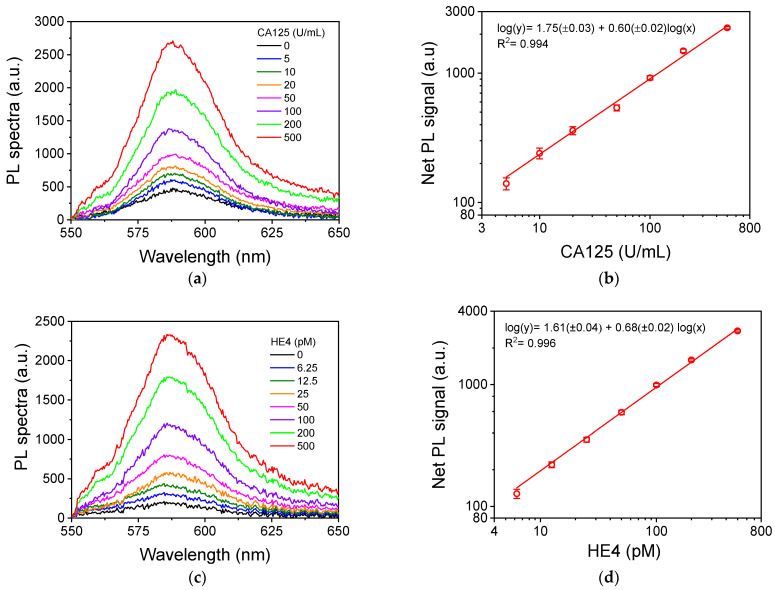
(**a**,**c**) Characteristic PL spectra received for (**a**) CA125 calibrators with concentrations from 0 to 500 U/mL and (**c**) HE4 calibrators with concentrations from 0 to 500 pM. (**b**,**d**) Typical calibration curves for (**b**) CA125 and (**d**) HE4. Each point is the mean value of 5 measurements from 3 replicate samples ± 3SD.

Using the optimum values for each immunoassay condition, the respective calibration curves were obtained, and the analytical characteristics of both immunoassays were determined. In Figure 7a,c, characteristic PL spectra corresponding to calibrators of CA125 and HE4, respectively, are illustrated, while the respective calibration curves are provided in Figure 7b,d. The equations of the calibration curves (also shown in the figures) were log(y) = 1.75(±0.03) + 0.60(±0.02)log(x) for CA125, with a coefficient of determination R^2^ = 0.994, and log(y) = 1.61(±0.04) + 0.68(±0.02)log(x) for HE4, with a coefficient of determination R^2^ = 0.996. The detection limit of each immunoassay was determined from the respective calibration curve equation as the concentration corresponding to a signal equal to 5 standard deviations of 10 values of the zero calibrator, while the limit of quantification was defined as the concentration corresponding to a signal equal to 10 standard deviations of 10 readings of the zero calibrator. More specifically, for the CA125 assay, the zero calibrator value was 460 ± 19.5 units, while that for the HE4 assay was 201 ± 17.5 units. Thus, the limit of detection and quantification were 2.5 and 5.0 U/mL for CA125, and 3.12 and 6.25 pM for HE4, respectively. The linear dynamic range extended up to 500 U/mL for CA125 and up to 500 pM for HE4. Thus, the assays for CA125 and HE4 determination have sufficient sensitivity and dynamic range to cover the concentration ranges of both healthy and ovarian cancer patients. It should be noted that the enzyme immunoassays that were developed in microtiter plates using the same antibodies and conditions presented the same detection sensitivity and linear dynamic range (Figures S3 and S4). Taking into account that in the case of an enzyme immunoassay the signal is considerably enhanced by the enzyme, the fact that the same detection sensitivity was achieved when the assays were performed on the nanostructured Ag substrates implemented in this work is indicative of the PL signal surface enhancement.

It is worth noting that the kits used for the determination of CA125 and HE4 (Affinity i Ca125 II and Affinity i HE4, Abbott Laboratories, Abbott Park, IL, USA) in an automated clinical analyzer have quantification limits of 1.4 U/mL and 20 pM, respectively, with linear dynamic ranges up to 1000 U/mL and 1500 pM, respectively. Compared to these assays, the assay developed for CA125 has a three-times-higher quantification limit, whereas the HE4 assay has a 3-times-lower quantification limit. Both assays have shorter linear dynamic ranges compared to those of the respective kits; however, encountering such high concentrations in human serum samples is highly unlikely.

To assess the reproducibility of the assays, control samples from commercially available kits (Affinity i Ca125 II Controls and Affinity i HE4 Controls, Abbott Laboratories, Abbott Park, IL, USA) were used. More specifically, to determine the intra-analytical repeatability, control samples were analyzed in triplicate on the same day and the coefficient of variation (CV) was determined, while for the estimation of the inter-analytical repeatability, the same control sera were analyzed in duplicate in immunochemical assays performed on four different days. The intra- and inter-assay CVs determined were less than 10 and 12%, respectively, for both assays. The accuracy of the assays was also tested by performing recovery tests. For this purpose, the controls with the lower concentration were spiked with known quantities of CA125 and HE4 and assayed prior to and after the addition. The results are presented in Table 1. As shown, the percent recovery values ranged from 83.3 to 117%, which is an indication of the assay accuracy [30].

The specificity of each assay against the other marker was tested by running the CA125 assay using the HE4 calibrators, and vice versa. The signals received for all CA125 and HE4 concentrations in the assays of HE4 and CA125, respectively, were similar to those obtained for the zero calibrator of each assay. Moreover, the calibration curves obtained for both markers with calibrators prepared either in assay buffer or in human serum were identical, indicating the absence of any interference from human serum.

### 3.4. Comparison with Literature Methods Based on Photoluminescence Detection

There are a few reports in the literature on the determination of CA125 and HE4 based on photoluminescence measurements with or without plasmonic signal enhancement. Thus, regarding CA125 determination with fluorescence-based sensors, a magnetic molecularly imprinted polymer made using an anti-CA125 antibody as a template was combined with Ni and Cd nanoclusters as labels, achieving an impressive detection limit of 50 μU/mL [31]. Fluorescence sensors employing aptamers as antigen-binding moieties have also been developed [32,33,34]. Thus, dual-color DNA–silver nanocluster–aptamer conjugates were applied for the simultaneous determination of carcinoembryonic antigen (CEA) and CA125 through salt-induced aggregation of gold nanoparticles, which resulted in lighting up the emitted fluorescence, leading to a detection limit for CA125 of 0.015 U/mL [32]. In another report, gold nanoparticles were modified with a dendrimer and then with an anti-CA125 antibody and were combined with carbon dots modified with an aptamer that binds CA125 to develop a homogeneous non-competitive assay based on fluorescence energy transfer from the carbon dots to gold nanoparticles [33]. A detection limit of 0.5 fg/mL was reported, which is difficult to correlate with the measurement unit usually employed to express CA125 concentration in clinical samples, U/mL. The fluorescence quenching effect of graphene oxide was also exploited through immobilization of a fluorescently labeled aptamer specific for CA125 and the measurement of emitted fluorescence upon incubation of graphene oxide with CA125 solution with a detection limit that was improved by 1000 times (from 50 to 0.05 U/mL) after treatment with a 5% formaldehyde solution to fixate the immobilized molecules onto the substrate [34]. Furthermore, two sensors for the detection of CA125 without the use of capture biomolecules have also been reported [35,36]. Both methods were based on fluorescence signal quenching upon incubation of the substrates with CA125, and the detection limits achieved were 1.45 and 0.66 U/mL, respectively. For the detection of HE4, an immunosensor that takes advantage of the metal-enhanced fluorescence effect when immunocomplexes were formed between antibody-modified carbon dots and antibody-modified silver nanoparticles was used, achieving a detection limit of 2.3 pM (57.5 pg/mL assuming a molecular weight of 25,000) [37]. In addition to methods developed for the detection of either CA125 or HE4, there are also literature reports on the simultaneous detection of CA125 and HE4 [29,38,39,40]. Thus, Yao et al. detected CA125 and HE4 simultaneously on microtiter wells with non-competitive immunoassays using different for each analyte label, achieving detection limits of 9.4 U/mL and 100 pM (1.25 ng/mL) for CA125 and HE4, respectively [29]. In another report, a nanostructured gold chip was employed as solid support for the development of an antibody microarray for the simultaneous detection of CA125, HE4, and osteopontin in combination with fluorescently labeled detection antibodies [38]. The detection limits achieved for CA125 and HE4 were 1.2 U/mL and 0.1 fmol/mL (2.5 pg/mL), respectively. Glass substrates were modified with graphene oxide nanoparticles, and four different capture antibodies (antibodies against CA125, HE4, CEA, and AFP) were immobilized onto discrete areas of this substrate by employing a microfluidic chip with several parallel channels [39]. Then, another fluidic chip was applied with a channel direction vertical to the first one, so each sample and the fluorescently labeled detection antibodies ran over the four capture ones. This configuration led to assays with detection limits of 0.01 U/mL for CA125 and 40 fM (1 pg/mL) for HE4. Finally, a lateral flow immunoassay strip for the simultaneous detection of CA125 and HE4 was realized using as labels carbon dots loaded on dendritic mesoporous silica nanoparticles [40]. The LOD of CA125 was 0.5 U/mL and that of HE4 was 2 pM (0.05 ng/mL). The detection limits achieved with the proposed sensor, although not the lowest compared to those reported in the literature, were more than adequate to detect both markers in healthy individuals and monitor ovarian cancer in patients undergoing chemotherapy. Moreover, the measurements in the current work were obtained with a low-cost optical set-up and conventional fluorescent labels, whereas some of the more sensitive sensors rely on quantum dots that have much higher quantum efficiency compared to conventional fluorescent labels and less background signal. Another advantage of the proposed sensor is the simplicity of nanostructured substrate fabrication, which can be readily performed in every chemical lab. However, for more reliable ovarian cancer detection, further work to develop a multianalyte scheme based on our approach is required. This requires the spatial confinement of the antibodies against the two analytes at specific positions on the substrate. A way to achieve this is by patterning the substrate so that SiNWs decorated with Ag aggregates are grown only in predefined positions. Such an approach could also facilitate assembly adhesion of microfluidics channels on the surfaces, which would allow the assays to be performed under flow, leading to lower detection limits and/or reduced assay duration.

## 4. Conclusions

We presented a method of detecting the CA125 and HE4 ovarian cancer biomarkers using a simple PL set-up and Ag-decorated Si nanowire substrates that can be easily fabricated in a few minutes with a simple etching/deposition process. The detection limits of CA125 and HE4 were 2.5 U/mL and 3.12 pM, respectively, with a linear dynamic range extending up to 500 U/mL for CA125 and 500 pM for HE4. The working ranges of both assays cover the concentrations encountered in both healthy individuals and ovarian cancer patients. While the current system uses a spectrometer, the PL signal detection requires only a photodiode and a laser-blocking long-pass filter. Thus, a lightweight and low-cost instrument can be effortlessly prepared and implemented along with the nanostructured substrates and assays developed for cost-effective ovarian cancer diagnosis and treatment monitoring.

## Figures and Tables

**Table 1:** CA125 and HE4 recovery experiments.

Analyte	Initial Concentration	Concentration Added	Concentration Determined	%Recovery
CA125 (U/mL)	40	30	65	83.3
		60	110	117
		90	125	94.4
HE4 (pM)	50	25	78	112
		50	145	90.0

## Data Availability

The data presented in this study are available on request from the corresponding author. The data are not publicly available due to privacy issues.

## Supplementary Materials

The following supporting information can be downloaded at: https://www.mdpi.com/article/10.3390/nano13243099/s1, Figure S1: SEM images of (a) Si nanowires decorated with Ag dendrites, (b) plain Si nanowires after removal of Ag dendrites, (c) Si nanowires decorated with Ag aggregates. The scale bar in all images is 100 nm; Figure S2: schematic of the in-house-developed optical set-up used for the PL measurements; protocol of CA125 enzyme immunoassay; Figure S3: characteristic CA125 enzyme immunoassay calibration curve. Each point is the mean of three replicates ± SD; protocol of HE4 enzyme immunoassay; Figure S4: characteristic HE4 enzyme immunoassay calibration curve. Each point is the mean of three replicates ± SD.
