# Supplementary material for: Cancer Marker Immunosensing through Surface-Enhanced Photoluminescence on Nanostructured Silver Substrates
Source: Nanomaterials (Basel). 2023 Dec 7;13(24):3099. doi: 10.3390/nano13243099 (PMC10745687; doi:10.3390/nano13243099)
Supplement: Supplementary file 1 [file nanomaterials-13-03099-s001.zip › nanomaterials-2714080-supplementary.pdf]

nanomaterials

Supplementary material

Cancer Markers Immunosensing through Surface-Enhanced Photoluminescence on Nanostructured Silver Substrates

Georgia Geka[1,2], Anastasia Kanioura[1], Ioannis Kochylas[3], Vlassios Likodimos[3], Spyros Gardelis[3], Anastasios Dimitriou[4], Nikolaos Papanikolaou[4], Kalliopi Chatzantonaki[5], Ekaterina Charvalos[5], Anastasios Economou[2], Sotirios Kakabakos[1], Panagiota Petrou[1,*]

[1] Immunoassays/Immunosensors Lab, Institute of Nuclear & Radiological Sciences & Technology, Energy & Safety, NCSR "Demokritos", 15341 Aghia Paraskevi, Greece
[2] Department of Chemistry, National and Kapodistrian University of Athens, University Campus, 15771 Zografou, Athens, Greece
[3] Section of Condensed Matter Physics, Department of Physics, National and Kapodistrian University of Athens, University Campus, 15784 Zografou, Athens, Greece
[4] Institute of Nanoscience & Nanotechnology, NCSR "Demokritos", 15341 Aghia Paraskevi, Greece
[5] Molecular Diagnosis Department, INVITROLABS S.A., 12251 Peristeri, Greece
* Correspondence: ypetrou@rrp.demokritos.gr; +30 210 6503819

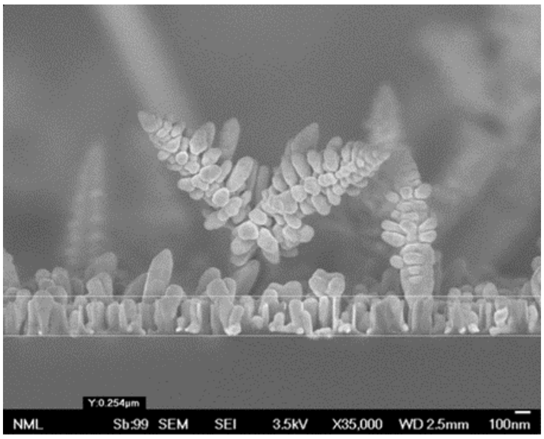

(a)

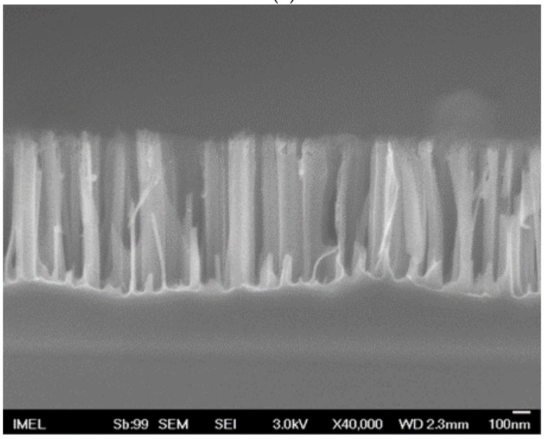

(b)

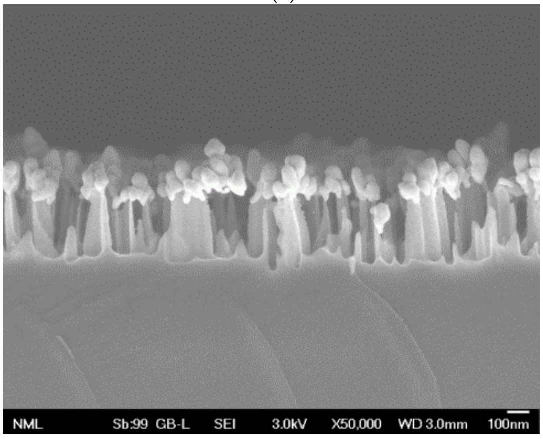

(c)

Figure S1. SEM images of: (a) Si nanowires decorated with Ag dendrites, (b) plain Si nanowires after removal of Ag dendrites, (c) Si nanowires decorated with Ag aggregates. The scale bar in all images is 100 nm.

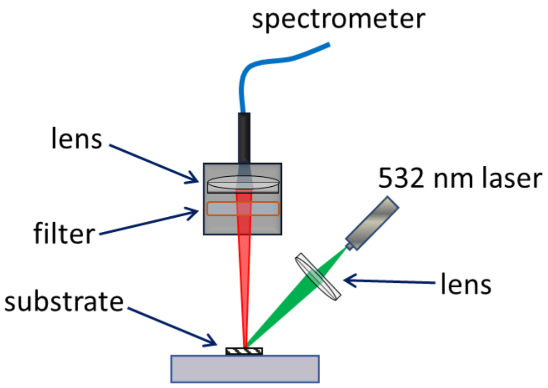

Figure S2. Schematic of the in-house developed optical set-up used for the PL measurements.

Protocol of CA125 enzyme immunoassay

Microtiter wells were incubated overnight at room temperature with 100 μL of a 5 μg/mL solution of anti-CA125 mAb (code 4601) in 10 mM Tris-HCl buffer, pH 8.25, 0.1 M NaCl. The wells were then washed twice with 300 μL of 10 mM Tris-HCl, pH 8.25, 9 g/L NaCl (washing solution 1), and blocked through incubation for 2 h with 300 μL of 0.1 M $NaHCO_3$ solution, pH 8.5, 10 mg/mL BSA. After washing the wells as previously, 50 μL of CA125 calibrators (0, 5, 10, 20, 50, 100, 200 and 500 U/mL) and 50 μL of a 2.5 μg/mL biotinylated anti-CA125 mAb solution (clone 4602) both prepared in 50 mM Tris-HCl buffer, pH 7.8, 5 mg/mL BSA, 9 g/L NaCl, 0.5 g/L NaN_3 were added per well and incubated for 1 h under shaking at room temperature. The wells were then washed 4 times with 300 μL of washing solution containing 0.5 mL/L Tween 20 (washing solution 2) and then 100 μL of a 250 ng/mL streptavidin-HRP conjugate solution in 50 mM phosphate buffer pH 7.0, 9 g/L NaCl, 10 mg/mL BSA were added and incubated for 30 min under shaking at room temperature. After washing as previously, 100 μL of HRP substrate (0.3 mL/L H_2O_2 and 0.0019 M ABTS in 0.1 M citrate-phosphate buffer, pH 4.5) were added per well and incubated for 30 min under shaking. The optical density at 405 nm was measured after 30 min of incubation under shaking using a VICTOR[3] 1420 Multilabel Counter (PerkinElmer).

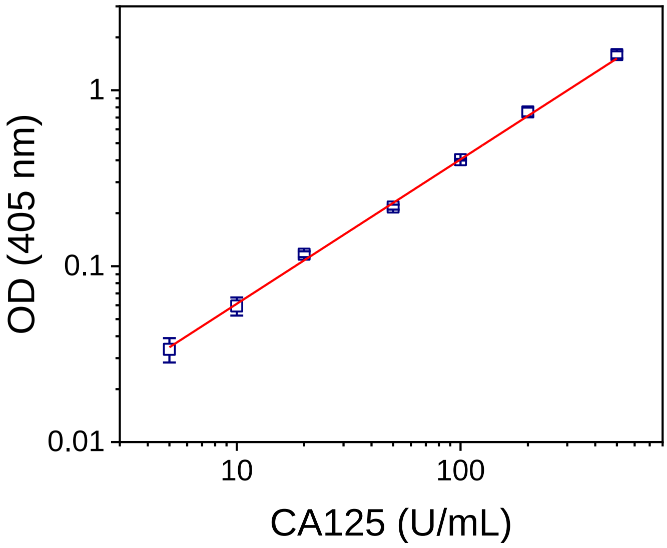

Figure S3. Characteristic CA125 enzyme immunoassay calibration curve. Each point is the mean of three replicates ± SD.

Protocol of HE4 enzyme immunoassay

Microtiter wells were incubated overnight at room temperature with 100 μL of a 5 μg/mL solution of anti-HE4 mAb (code 4501) in 50 mM carbonate buffer, pH 9.2. The wells were then washed twice with 300 μL of 10 mM phosphate buffer saline buffer (PBS), pH 7.4, (washing solution 1) and blocked through incubation for 2 h with 300 μL of 0.1 M $NaHCO_3$ solution, pH 8.5, 10 mg/mL BSA. After washing the wells as previously 50 μL of HE4 calibrators (0, 0.15, 0.30, 0.6, 1.25, 2.5 and 5.0 ng/mL) and 50 μL of a 2.5 μg/mL biotinylated anti-HE4 mAb solution (clone 4505) both prepared in 50 mM PBS, pH 7.4, 10 mg/mL BSA, 0.5 mg/mL NaN_3 were added per well and incubated for 1 h under shaking at room temperature. The wells were then washed 4 times with 300 μL of washing solution containing 0.5 mL/L Tween 20 (washing solution 2) and then 100 μL of a 250 ng/mL streptavidin-HRP conjugate solution in 50 mM phosphate buffer pH 7.0, 9 g/L NaCl, 10 mg/mL BSA were added per well and incubated for 30 min under shaking at room temperature. After washing as previously, 100 μL of HRP substrate (0.3 mL/L H_2O_2 and 0.0019 M ABTS in 0.1 M citrate-phosphate buffer, pH 4.5) were added per well and incubated for 30 min under shaking. The optical density at 405 nm was measured after 30 min of incubation under shaking using a VICTOR^3 1420 Multilabel Counter (PerkinElmer).

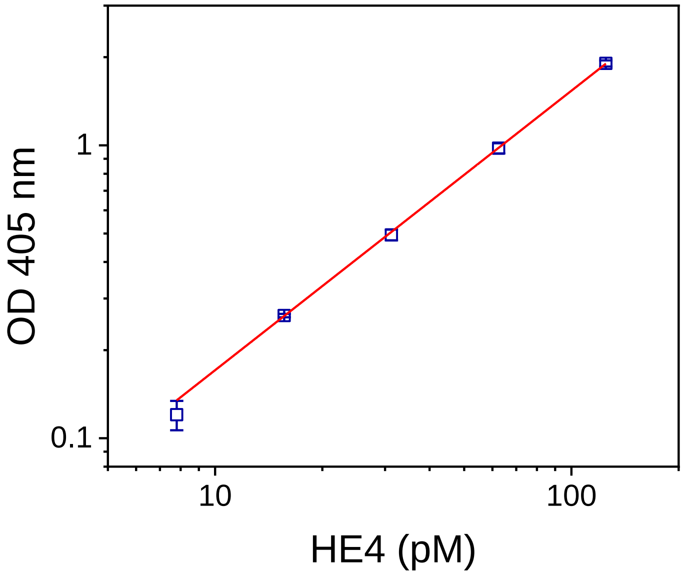

Figure S4. Characteristic HE4 enzyme immunoassay calibration curve. Each point is the mean of three replicates ± SD.
